# A New coumarin from *Citrus paradisi* Macf.

**DOI:** 10.4103/0250-474X.44608

**Published:** 2008

**Authors:** Meera, S. B. Kalidhar

**Affiliations:** Department of Chemistry, Haryana Agricultural University, Hisar-125 004, India

**Keywords:** *Citrus paradisi*, Rutaceae, coumarin, friedelin, β-sitosterol, limonin, cordialin B

## Abstract

Phytochemical examination of the peel of grapefruit resulted in the isolation of five compounds namely friedelin, β -sitosterol, 7(3’,7’,11’,14’-tetramethy)pentadec-2’,6’,10’-trienyloxycoumarin, limonin and cordialin B. These compounds have been characterized on the basis of spectral data, and 7(3’,7’,11’,14’-tetramethy)pentadec-2’,6’,10’-trienyloxycoumarin is a hitherto unreported compound.

*Citrus paradisi* commonly known as grapefruit and *pahari nimbu* belongs to the family Rutaceae. It is synonymous with *C. decumana* var. racemosa, *C. decumana* var. paradisi Nichols and *C. racemosa* Macf. The juice of the grapefruit is used for building up resistance to common cold and wound infections^1^. There is report of only one compound i.e. 6,7-dimethoxycoumarin^2^ from grapefruit peel. In view of existing scanty data, the present study was undertaken (fig 1).

**Fig. 1:**
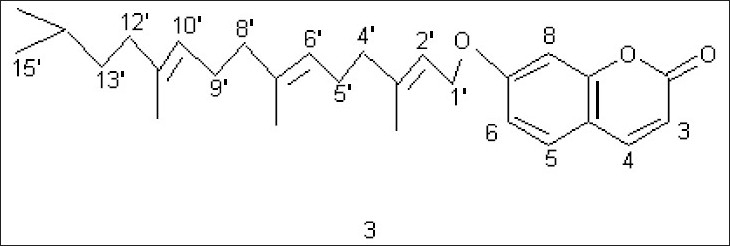
Structure for the coumarin isolated from *Citrus paradisi*

Melting points were determined on Ganson Electrical Melting Point Apparatus. IR spectra were recorded on Hitachi 570 Infrared Spectrophotometer using KBr. ^1^H NMR spectra were recorded on Brucker AC-300F 300 MHz NMR Spectrophotometer using TMS as an internal standard. Chemical shifts are given in δ (ppm) and CDCl_3_ was used as a solvent for recording NMR spectra. Fruits of *C. paradisi* (10 kg) were procured from Landscape, HAU, Hisar.

The peel of the fruits was removed and air dried. The peel was then refluxed with hot methanol for 6 h and the process was repeated 4 times. Extractives were concentrated on water bath under reduced pressure and the viscous mass thus obtained was mixed with silica gel (60-120 mesh), dried on water bath and subjected to column chromatography. Petroleum ether was used as a solvent in packing the column. Five compounds were isolated.

Compound A crystallized from methanol, 25 mg, mp 268° (literature mp 267.3-269.5°)^3^. It gave pale brown color on reaction with Ac_2_ O/H_2_ SO_4_; IRν_max_ (KBr, cm^-1^): 1720; ^1^H NMR (δ, CDCl_3_): 2.25-1.20 (25 H, m, 11*CH_2_ and 3*CH), 1.17 (3 H, s, CH_3_), 1.04 (3H, s, CH_3_), 1.00 (6H, s, 2*CH_3_), 0.95 (3 H, s, CH_3_), 0.86 (6H, s, 2*CH_3_), 0.72(3H, s, CH_3_); GCMS: 426 (M^+^). This data settled compound A to be friedelin (1)^3^.

Compound B crystallized from methanol, 20 mg, mp 136° (literature mp 136-137°)^4^. It responded to Liebermann-Burchard Reaction. IRν_max_ (KBr, cm^-1^): 3427; ^1^H NMR (δ, CDCl_3_): 5.34 (1H, br, H-6), 3.51 (1H, m, H-3), 2.28-1.13 (29H, m, 11*CH_2_, 7*CH), 0.92 (6H, s, 2*CH_3_), 0.83 (3H, s, CH_3_), 0.80 (3H, s, CH_3_), 0.78 (3H, s, CH_3_), 0.68 (3H, s, CH_3_); GCMS: 414 (M+). This data confirmed compound B to be β-sitosterol (2)^5^ using a direct comparison.

Compound C (3) crystallized from methanol, 50 mg, mp 70°. IRν_max_ (KBr, cm^-1^): 1700; ^1^H NMR (δ, CDCl_3_): 7.64 (1H, d, *J* 10 Hz, H-4), 7.36 (1H, d, *J* 7.5 Hz, H-5), 6.82 (2H, m, H-6, H-8), 6.25 (1H, d, *J* 10 Hz, H-3), 5.50 (1H, 1H, t, *J* 7.0 Hz, H-2’), 5.10 (2H, t, *J* 7.0 Hz, H-6’, H-10’), 4.61 (2H, d, *J* 7.0 Hz, 2*H-1’), 2.34 (4H, m, 2*H-5’, 2*H-9’), 2.10 (6H, t, *J* 7.0 Hz, 2*H-4’, 2*H-8’, 2*H-12’), 1.75 (3H, s, CH_3_), 1.72 (3H, s, CH_3_), 1.65 (3H, s, CH_3_), 1.30-0.86 (9H, m, 2*CH_3_, CH_2_, CH); GCMS: 422 (M^+^).

Compound D crystallized from methanol, 40 mg, mp 297 (literature mp 298)^3^. IRν_max_ (KBr, cm^-1^): 1758,1708; ^1^H NMR (δ, CDCl_3_): 7.41 (2H, m, H-2’, H-5’), 6.30 (1H, m, H-3’), 5.46 (1H, s, OCHCOO), 4.77-4.47 (2H, m, 2*OCH), 4.02 (2H, s, OCH_2_), 2.98-1.77 (10H, m, 4*CH_2_, 2*CH), 1.29 (3H,s, CH_3_), 1.17 (6H, s, 2*CH_3_), 1.07 (3H,s, H_3_); GCMS: 470 (M^+^). The data proposed compound D to be limonin (4)^3^.

Compound E crystallized from methanol, 50 mg, mp 114° (literature mp 114-115°)^3^. It responded to Liebermann-Burchard Reaction. IRν_max_(KBr, cm^-1^): 3358; Acetate: compd.+Ac_2_O+Py; ^1^H NMR (δ, CDCl_3_): 5.34 (1H, m, =CH), 4.28 (1H, m, CH-O), 4.10 (1H, m, CH-O), 3.65(1H, m, CH-O), 3.39(1H, m, CH-O), 2.32-1.08 (20H, m, 8*CH_2_, 4*CH), 2.08 (3H, s, OAc), 2.04 (3H, s, OAc), 2.01 (3H, s, OAc), 2.00 (3H, s, OAc), 1.23 (3H, s, CH_3_), 1.10 (3H, s, CH_3_), 0.98 (3H, s, CH_3_), 0.92 (3H, s, CH_3_), 0.88 (3H, s, CH_3_), 0.86 (3H, s, CH_3_), 0.67 (3H, s, CH_3_), GCMS: 490 (M^+^). A comparison with literature data^3^ suggested this compound to be cordialin B (5).

Compound C showed fluorescence under UV indicating it to be a coumarin. The IR spectrum of the compound exhibited the presence of a carbonyl group at 1700 cm^-1^ which was a further support towards the coumarin nucleus. MS suggested its molecular mass to be 422. ^1^H NMR of the compound in CDCl_3_ showed a doublet at δ 7.64 (*J* 10 Hz) which was typical of H-4 of a coumarin. Another doublet was observed at δ 7.36 (*J* 7.5 Hz) which could be H-5 of a coumarin. There was a multiplet at δ 6.82 for two protons which represented H-6 and H-8 of the nucleus. A doublet at δ 6.25 (*J* 10 Hz) was assignable to H-3. A triplet at δ 5.50 (*J* 7.0 Hz) could be H-2’ of an aliphatic chain attached at C-7 of the nucleus. Another triplet at δ 5.10 (*J* 7.0 Hz) for two protons could be due to H-6’ and H-10’. A doublet at δ 4.61 (*J* 7.0 Hz) was assignable to 2 protons at C-1’. A multiplet at δ 2.34 for 4 protons could be 2*H-5’ and 2*H-9’. A triplet at δ 2.10 (*J* 7.0 Hz), representing 6 protons, could be 2*H-4’, 2*H-8 and 2*H-12’. Three singlets at δ 1.75, 1.72 and 1.65, each for 3 protons, could be due to 3 methyls, one each at C-3’, C-7’ and C-11’. A multiplet in the range 1,30-0.86 for 9 protons could be 2 methyls, one methylene and one methine. The data suggested the compound to be 7(3’,7’,11’,14’-tetramethy)pentadec-2’,6’,10’-trienyloxycoumarin (3) which is a hitherto unreported compound.
